# A systematic approach for the identification of novel, serologically reactive recombinant Varicella-Zoster Virus (VZV) antigens

**DOI:** 10.1186/1743-422X-7-165

**Published:** 2010-07-20

**Authors:** Maria G Vizoso Pinto, Klaus-Ingmar Pfrepper, Tobias Janke, Christina Noelting, Michaela Sander, Angelika Lueking, Juergen Haas, Hans Nitschko, Gundula Jaeger, Armin Baiker

**Affiliations:** 1 Max von Pettenkofer-Institute, Virology, Munich, Germany; 2 Mikrogen GmbH, Neuried, Germany; 3 Protagen AG, Dortmund, Germany; 4 University of Edinburgh, Division of Pathway Medicine, Edinburgh, UK

## Abstract

**Background:**

Varicella-Zoster virus causes chickenpox upon primary infection and shingles after reactivation. Currently available serological tests to detect VZV-specific antibodies are exclusively based on antigens derived from VZV-infected cells.

**Results:**

We present a systematic approach for the identification of novel, serologically reactive VZV antigens. Therefore, all VZV open reading frames were cloned into a bacterial expression vector and checked for small scale recombinant protein expression. Serum profiling experiments using purified VZV proteins and clinically defined sera in a microarray revealed 5 putative antigens (ORFs 1, 4, 14, 49, and 68). These were rearranged in line format and validated with pre-characterized sera.

**Conclusions:**

The line assay confirmed the seroreactivity of the identified antigens and revealed its suitability for VZV serodiagnostics comparable to commercially available VZV-ELISA. Recombinant ORF68 (gE) proved to be an antigen for high-confidence determination of VZV serostatus. Furthermore, our data suggest that a serological differentiation between chickenpox and herpes zoster may be possible by analysis of the IgM-portfolio against individual viral antigens.

## Background

The Varicella-Zoster virus (VZV) is a member of the neurotropic alphaherpesvirus subfamily of the Herpesviridae. VZV causes varicella (chickenpox) during primary infection and may cause herpes zoster (shingles) as secondary disease after reactivation from latency.

Varicella can be considered as a harmless childhood disease. However, severe outcomes in the elderly, immunocompromised or resulting from congenital infection of the fetus or newborn are dreaded [1]. A live attenuated vaccine against varicella is available since 1995 and in Germany officially recommended since 2004 for vaccination of children in their second year of life. The introduction of universal varicella vaccination has substantially reduced varicella related morbidity and mortality [2,3]. Varicella vaccine was originally administered as a single dose, but this recommendation was modified in favour of a two dose regimen due to the occurrence of several breakthrough varicella infections [4-6]. Breakthrough varicella may occur months to years after immunization and is caused by wild-type VZV as a result of vaccine failure [7]. Vaccine failure is divided into two types. Primary vaccine failure occurs when no measurable immune response is elicited following vaccination, leaving the vaccinee susceptible to the disease. Secondary vaccine failure occurs when the immune response vanishes over time, leaving the vaccinee with a degree of susceptibility to the disease [2,8]. Waning of varicella immunity is of particular public health interest, since it may result in an increased susceptibility later in life, when the risk of severe complications may be greater than during childhood. Two main reasons are discussed for the phenomenon of waning varicella immunity, one being the decreased immune response and immunological memory elicited by the attenuated varicella, the other one being the reduction in (natural) exogenous booster exposures to VZV as a consequence of systematic mass vaccination programmes and the reduced circulation of this virus in the human population [2,8].

One major goal of VZV-specific laboratory diagnosis is the identification of immunological markers that correlate with protection against varicella. Such markers are extraordinarily important, since the diagnosis of susceptibility to the disease implies therapeutic consequences as for example active or passive immunization within certain person groups [6]. It is generally accepted that the presence of VZV-specific antibodies within immunocompetent persons serves as an immune correlate of protection, indicating immunity towards varicella disease. VZV-specific antibodies are directed against a variety of different viral antigens including glycoproteins (gps) as well as regulatory and structural proteins or viral enzymes [9]. Antibodies of special interest are those directed against VZV glycoprotein E (gE), since this viral protein has been shown to be the most abundant and immunogenic of all VZV gps eliciting both, the formation of neutralizing antibodies and the mediation of cellular cytotoxicity [10,11]. The relative importance of the individual protein-specific antibodies in prevention of reinfection, however, is not completely understood.

In this work we present a systematic strategy for screening and identification of novel, serologically reactive VZV antigens based on recombinant, bacterially expressed and purified VZV proteins (see Additional file 1). For this purpose, all 71 known VZV open reading frames (ORFs) were recombinatorially cloned into bacterial expression vectors. A systematic small scale protein expression and purification study in *E. coli *Rosetta (DE3) revealed, that ~35.2% of all VZV proteins could be expressed recombinantly, and ~25.4% of all VZV proteins could be purified in 96 well format. When we performed serum profiling experiments with clinically defined VZV patient sera in a microarray format, ~27.8% of the purified VZV proteins could be identified as putative serological marker antigens. The respective recombinant antigens were purified in large scale as described by Soutscheck et al. [12], rearranged in line assay format and validated with a number of pre-characterized serum samples. These validation data confirmed the seroreactivity of the identified marker antigens and revealed the suitability of the recombinant line assay for VZV serodiagnostics.

## Results

### Serum profiling experiments in microarray format

24 VZV proteins could be expressed in small-scale in *E. coli *Rosetta (DE3) but only 18 of them (75%) could be purified by means of Ni-NTA columns under these conditions. Respective 18 purified VZV antigens, namely: ORFs 1, 4, 9a, 14, 16, 18, 20, 32, 33.5, 39, 43, 49, 56, 60, 61, 62, 63 and 68 were spotted onto a microarray with a capacity for 34 spots (*recom*Dot system, Mikrogen). For control reasons we additionally spotted a VZV lysate (Virion, Serion, Germany). All antigens were spotted in duplicate.

For IgG monitoring, the microarrays were probed with clinically defined sera from acute chicken pox (n = 4), acute zoster (n = 9), and serologically defined VZV-IgG/IgM negative control sera (n = 5). The VZV lysate was nonreactive with all negative control sera and reactive with 13 clinically defined sera. Three of the recombinant antigens (ORFs 32, 33.5 and 63) reacted positive with one negative control serum. All other antigens were negative or inconclusive with all negative sera. ORF68 was positive with 12 (plus 1 inconclusive) out clinically defined sera tested. ORF1 reacted with one negative control sample. ORF49 was negative within all negative control samples but reacted with 6 out of 9 zoster patients. All other VZV antigens did not show any tendency towards one of the respective patient groups (data not shown).

When screening the same sera for IgM reactivity, the VZV lysate was reactive with 6 clinically defined sera. Only 4 of the recombinant VZV antigens were IgM reactive with at least one clinically defined serum: ORF4 (n = 1), ORF9a (n = 1), ORF20 (n = 4), and ORF68 (n = 1) and were not reactive in any negative sera tested. The reactivities of ORF9a and ORF20 were slightly above the cutoff level, those of ORF4 and ORF68 were significantly higher (data not shown).

According to these results, we selected ORFs 1, 4, 14, 49 and 68 for recloning, reexpression and repurification in large scale in order to apply these antigens onto a line assay (recomLine VZV).

### Evaluation of the identified VZV antigens by a line assay

#### VZV IgM and IgG reactivities in clinically defined sera

**Figure 1:**
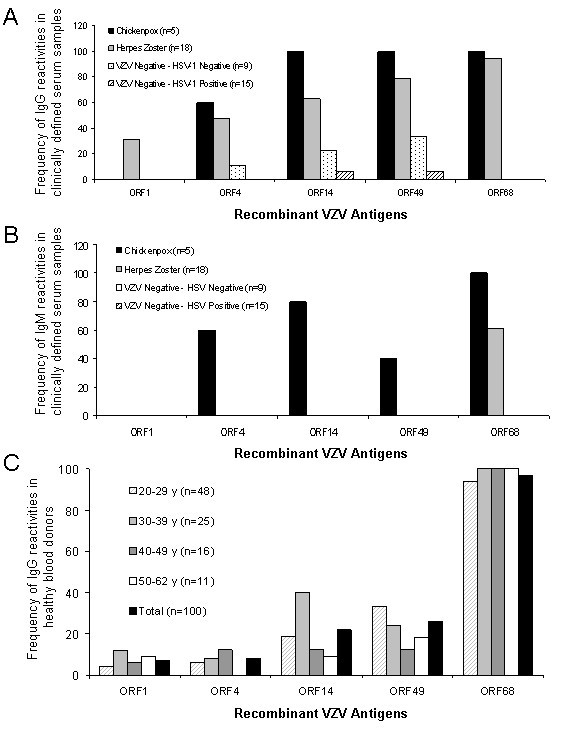
A) Frequency of IgG reactivity of clinically defined samples and serologically defined VZV-IgG/IgM negative samples, (*) as assayed by Enzygnostic wcELISA, to selected recombinant VZV antigens . B) Frequency of IgM reactivity of clinically defined samples and serologically defined VZV-IgG/IgM negative samples, (*) as assayed by Enzygnostic wcELISA, to selected recombinant VZV antigens. C) Frequency (%) of reactive serum samples (IgG) against specific VZV antigens in a panel of 100 blood donors grouped according to age. Single VZV antigen bands were considered as positive when their intensity was higher than the cut-off band control.

Nitrocellulose strips were coated with the selected VZV antigens and tested with clinically characterized sera from acute chickenpox (n = 5), acute zoster (n = 18) and serologically defined VZV-IgG/IgM negative control sera (n = 24) for the presence of IgG and IgM antibodies (Table 1). All clinically defined sera were additionally characterized by VZV IgG/IgM wcELISA (see Additional file 2). In our recomLine VZV-IgG assay none of the negative control sera was reactive against recombinant ORFs 1 and 68 (Figure 1A). However, ORFs 4, 14, and 49 were cross-reactive with IgG in some VZV-IgG/IgM negative serum samples. No correlation between cross-reactivity and HSV-IgG status could be observed (Figure 1A, see Additional file 3). Interestingly, ORF1 was only reactive with IgG of zoster sera (Figure 1A), which may be a hint pointing to ORF1 as a possible zoster marker candidate. In the recomLine VZV-IgM assay, none of the negative control serum samples was reactive with any recombinant antigen (Figure 1B). Interestingly, chickenpox and herpes zoster patients could be characterized by their IgM-portfolio against the various recombinant proteins. Whereas sera derived from chickenpox patients exhibited the tendency to react with several recombinant proteins (ORFs 4, 14, 49 and 68), sera derived from herpes zoster patients only reacted with ORF68 or showed no reactivity towards any recombinant protein (Figure 1B, see Additional file 2). This indicates that a serological differentiation between chickenpox and herpes zoster may be possible by analysis of the IgM-portfolio against individual viral antigens. The VZV-IgG detection limit of our novel recomLine VZV has been determined as 250 mIU/ml by using two-fold serial dilutions of a WHO VZV standard (50 IU/ml) reagent. The WHO VZV standard reagent exhibited only reactivity against ORF68, but not against other antigens (data not shown).

**Table 1:** Validation of the recomLine VZV with clinically defined sera

Assay	Result	Clinically defined sera				**Serologically defined VZV-IgG/IgM Neg sera**^**d**^	
				
		Chickenpox		Zoster			
		
		IgG	IgM	IgG	IgM	IgG	IgM
**ELISA**	**Pos **^a^	5	5	18	9	0	0
	**Inc **^b^	0	0	0	0	0	0
	**Neg **^c^	0	0	0	9	24	24

**RecomLine VZV**	**Pos**	5	5	17	11	0	0
	**Inc**	0	0	1	0	0	0
	**Neg**	0	0	0	7	24	24

**NOTE**. Data are no. of serum samples.

^a ^Pos: positive; ^b^Inc: inconclusive; ^c^Neg: negative, ^d^: as assayed by Siemens Enzygnost VZV IgG/IgM wcELISA.

RecomLine VZV strips were considered as positive when the intensity of the ORF68 band was higher than the cut-off band control.

#### VZV IgM and IgG reactivities in healthy donor sera

In a second series of experiments, we tested 100 samples of healthy blood donors using the standard wcELISA and our newly developed recomLine VZV (Table 2). We found that the diagnostic potential of the recomLine VZV is comparable to the Enzygnost wcELISA. When the antigens were analyzed individually, the frequencies of the reactivities varied from 7% (ORF1) to 97% (ORF68) among the healthy blood donors (Figure 1C). Due to the unweighted n for each group, we could not determine if there is a correlation between age and the reactivity to a certain VZV antigen. When testing IgM antibodies, we could only find reactivities against ORF14, ORF49 and ORF68 in five, two and three out of 100 tested samples, respectively (data not shown).

**Table 2:** Comparison of the recomLine VZV prototype with wcELISA in healthy blood donors

Result	wc ELISA		recomLine VZV	
	
	IgM	IgG	IgM	IgG
Pos^a^	1	95	1	97
Inc^b^	4	3	0	0
Neg^c^	95	2	99	3
**Specificity**	Gold standard		98.08^d ^- 100.0^e ^%	
**Sensitivity**			95.15^e ^- 97.96^d ^%	

**NOTE**. Data are no. of serum samples unless otherwise indicated.

^a ^Pos: positive

^b ^Inc: inconclusive

^c ^Neg: negative

To calculate sensitivity and specificity, the inconclusive results were grouped as ^d^negative or as ^e^positive.

RecomLine VZV strips were considered as positive when the intensity of the ORF68 band was higher than the cut-off band control.

### Time course of VZV seroconversion - a case report

**Figure 2:**
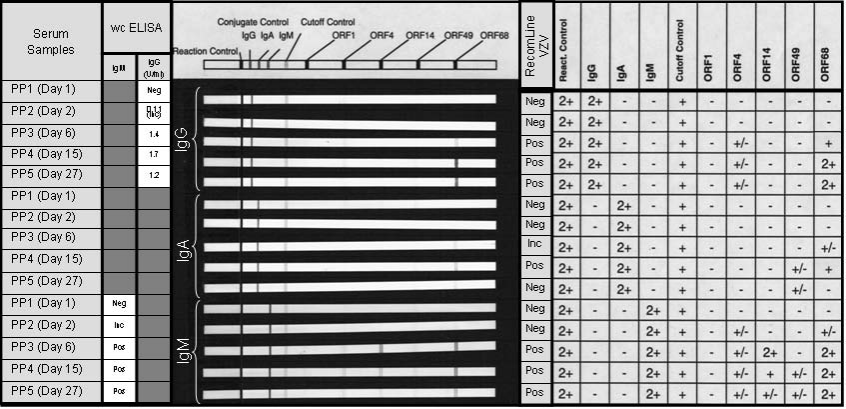
**Time course of seroconversion of an adult suffering a primary chicken-pox infection as determined by a VZV-Line assay, wcELISA and gpELISA **. Single VZV antigen bands were considered as positive when their intensity was higher than the cut-off band control.

In addition, we analyzed a time course of seroconversion of a person with primary varicella infection. The immunocompetent patient presented a typical varicella skin rash and fever and seroconverted in IgG and IgM as detected by wcELISA and the recomLine VZV assay between days 2 and 6 after the first onset of symptoms (Figure 2). ORF1 did not react with neither IgG, IgA nor IgM antibodies at any time point, whereas ORF4 reacted very weakly with IgG and IgM. ORF68 was the only antigen that reacted with the three types of antibodies tested (IgM, IgG and IgA) and reached the highest intensity at day 15. IgA was clearly less reactive than IgM or IgG and the levels of anti-ORF68 declined below the cut-off level by day 27. ORF14 and ORF4 only reacted with IgM antibodies peaking around day 6 and 15 after the first onset of symptoms, respectively and declining at day 27 (Figure 2).

## Discussion

Virtually all currently available commercial tests for the detection of VZV-specific antibodies, i.e. *Fluorescent antibody to membrane antigen *(FAMA), *latex agglutination *(LA) and *ELISA*, are exclusively based on whole antigens or antigen extracts derived from VZV-infected cell culture and therefore lack the ability to differentiate between antibodies directed against individual VZV proteins. FAMA can be considered as the gold standard test and has been shown to correlate best with susceptibility to and protection against varicella. The FAMA test principle is indirect immunofluorescence microscopy using VZV-infected cells as antigen [13]. This strategy optimally preserves the conformational structure of surface membrane proteins, being responsible for the extraordinary sensitivity of this assay. FAMA titers of ≥ 1:4 strongly correlate with protection from varicella after household exposure [14]. However, the FAMA procedure is labor-intensive, needs considerable experience in handling VZV and cannot be automated.

LA, based on latex particles coated with extracted VZV gps, has been reported to correlate well with FAMA [4,8,15]. Disadvantages of LA are, that test interpretation requires experience in reading agglutination, that it cannot distinguish between various antibody subclasses (e.g. IgG and IgM), and it is not amenable to automation. Furthermore, false-negative results due to prozone formation, and false-positive results have been described [16,17].

ELISA based tests can be classified according to the kind of VZV-antigens used. Whole cell ELISA (wcELISA) tests, the majority of commercially available VZV-ELISA tests, use whole lysates of VZV-infected cells as antigen, whereas the more sensitive glycoprotein ELISA (gpELISA) tests utilize VZV-gp extracts [15]. The original gpELISA method has been developed by Merck for extensive studies of children immunized with the Varivax Oka vaccine [18,19] and is not commercially available. However, similar gpELISA tests with high sensitivity have been introduced by different companies, as e.g. Virion/Serion and Ridascreen, recently [20,21].

For the development of novel serological tests to detect VZV-specific antibodies it is important to keep in mind, that assays with low sensitivity may result in unnecessary vaccinations, which are costly to the public health system, and assays with low specificity are prone to produce false-positive results, mistakenly depriving persons at risk of varicella of an indicated therapy. After establishing of mass vaccination programmes within many industrialized countries, primary and secondary vaccine failures have occurred in parallel with the observation of waning immunity to VZV primary disease after varicella vaccination. Hence, the identification of novel varicella immune correlates of protection and the consequently development of novel serological tests is strongly desirable [15].

Here we present a pipeline for the screening of novel serological markers of (in this case: VZV) infection. By using this systematic approach we could identify five antigens (ORFs 1, 4, 14, 49 and 68) that were serologically reactive as recombinant, bacterially expressed proteins. It is noteworthy, that all identified antigens are components of the mature VZV virion: ORF14 and ORF68 are both glycoproteins anchored in the viral envelope [10]; ORF1 is a tail-anchored membrane protein facing the tegument with its N-terminus [22] and ORF4 and ORF49 are both viral tegument proteins [23,24]. Furthermore, ORF4 has also been identified as a novel target protein for persistent VZV specific CD4^+ ^T cells, which may be involved in the control of VZV reactivation [25]. ORF68 has already been described as highly immunogenic VZV protein, eliciting both, humoral and cellular immune responses [11,26]. Recently, recombinant ORF68 has been utilized for the development of serological herpesvirus microarray [27]. With our newly developed recomLine VZV, we could further confirm the suitability of ORF68 as a highly confident marker for VZV serostatus. It needs to be further investigated if recombinant ORF68 alone may serve as antigen for the efficacy control for varicella vaccination. In contrast, the novel identified antigens (ORFs 1, 4, 14 and 49) showed other patterns of reactivity, which should be further investigated in order to find possible correlations with different clinical entities of VZV infection or VZV related immunity. According to the analysis of 23 clinically defined sera, anti-ORF1 IgG may be a zoster marker candidate (Figure 1A). As suggested by the analysis of these clinically defined sera (Figure 1B) and by our time course of VZV seroconversion after primary infection (Figure 2), the portfolio of IgM antibodies against individual recombinant antigens may enable the serological differentiation between chickenpox (ORFs 4, 14, 49, and 68) and herpes zoster (only ORF68). The suitability of in vitro transcribed and translated ORF14 using a self-assembled protein microarray (NAPPA) for serodiagnostics has also been proposed in parallel to this work by Ceroni et al. [28]. However, the detailed significance of detectable IgG and IgM antibodies against the various recombinant antigens, within the status of VZV infections needs to be further investigated.

## Conclusions

We present a systematic approach for the identification of novel, serologically reactive markers of infection (in this case: VZV). The knowledge about the VZV serostatus is extraordinarily important for immunocompromised patients and pregnant women in order to take prophylactic and/or therapeutic measurements after VZV exposure [29]. The recomLine VZV assay based only on the ORF68 recombinant protein is a reliable, relatively fast (approximately 2.5 h), easy to handle and interpret test, which can be used outside of traditional laboratory settings such as clinics, community outreach centres and physician practices to check for VZV-IgG serostatus.

Furthermore, as it is automatable it could also be used for large screenings in e.g. epidemiological studies. The relevance of the further identified antigens will be further investigated with a larger number of specimens.

## Methods

### Recombinatorial cloning of VZV ORFs

The nucleotide sequences of all 71 VZV ORFs were obtained from the ncbi http://www.ncbi.nlm.nih.gov/. BP recombination reactions of VZV ORFs into pDONR207 (Invitrogen, Germany) were performed as described earlier [30]. Briefly, all 71 VZV ORFs with *attB*-sites were amplified by nested PCR, using the "first round PCR" (VZV-ORF-specific) primer set: *VZV-ORF-forward*: 5'-AAAAAGCAGGCTCCGCC(18-22 ORF-sequence specific nucleotides including start codon)-3' and *VZV-ORF-reverse*: 5'-AGAAAGCTGGGTC(18-22 ORF-sequence specific nucleotides including stop codon)-3' and the "second round PCR" (one-for-all) primer set: *One-for-all-forward: *5'-GGGGACAAGTTTGTACAAAAAAGCAGGCT-3' and *One-for-all-reverse: *5'-GGGGACCACTTTGTACAAGAAAGCTGGGTC-3'. PCR products containing VZV-ORFs and functional *attB*-sites were gel purified using the QIAquick Gel Extraction Kit (Qiagen, Germany) and recombinatorially cloned into the *attP*-sites of pDONR207 using BP-clonase II enzyme mix (Invitrogen, Germany) according to the manufacturers' instructions. BP reactions were incubated at room temperature over night and subsequently transformed into chemically competent *E. coli *DH5α. Plasmid DNA of individual colonies grown on LB-plates supplemented with 12.5 μg/ml gentamycin (Invitrogen, Germany) was isolated using the QIAprep Spin Miniprep Kit (Qiagen, Germany) and the integrity of the resulting pENTR207-VZV-ORF vectors was verified by *Ban*II (New England Biolabs, Germany) restriction analysis and forward sequencing (AGOWA, Germany).

LR recombination reactions using LR-clonase II enzyme mix (Invitrogen, Germany) were performed according to the manufacturers' instructions. Briefly, pENTR207-VZV-ORF vectors containing VZV-ORFs flanked by *attL*-sites were recombinatorially cloned into the *attR*-sites of the customized vector pETG-A-His-N-[rfB]. The latter vector has been constructed by insertion of a customized cassette consisting of 5'-*Nhe*I-*Hind*III-ATG-[RGS-His-tag]-*EcoR*V-[ccdB/CmR(rfB)]-*EcoR*V-*Xba*I-*Sal*I-3' into the backbone of the bacterial expression vector pET-22b(+) (Novagen, Germany). LR clonase reactions were incubated at 37°C for 2 h and subsequently transformed into chemically competent *E. coli *DH5α. Plasmid DNA of individual colonies grown on LB-plates supplemented with 100 μg/ml ampicillin (Sigma-Aldrich, Germany) was isolated as described above and the integrity of the resulting pETG-A-His-N-VZV-ORF vectors was verified by *Hind*III/*Xba*I (New England Biolabs, Germany) restriction analysis.

### Small scale expression and purification of His-tagged VZV proteins

Systematic small scale protein expression and purification was performed as described recently [31] with minor modifications. Briefly, all 71 constructed pETG-A-His-N-VZV-ORF vectors were transformed individually into chemically competent *E. coli *Rosetta (DE3). Transformed bacteria were selected on LB-plates supplemented with 100 μg/ml ampicillin (Sigma-Aldrich, Germany). Pools of ten colonies per LB-plate were picked and resuspended in freezing media (LB-media supplemented with 15% glycerol). The respective 71 pools of transformed bacteria were stored in a 96-well (round bottom) plate (Genetix, UK) at -80°C until further analysis.

For analysis of recombinant protein expression, all steps were performed in 96-well format using a Liquidator^96 ^manual 96-channel pipetting tool (Steinbrenner, Germany). The 96-well glycerol culture plate was thawed and used for inoculation of 1.5 ml LB-media supplemented with 100 μg/ml ampicillin (Sigma-Aldrich, Germany) in a 96 (round bottom) deep well block (Qiagen, Germany). The latter block was incubated in a bacterial shaker at 37°C over night. For induction of recombinant protein expression, 1.4 ml LB-media were inoculated in a fresh 96 deep well block with 100 μl of the latter over night culture and incubated in a bacterial shaker for 3 h at 37°C before addition of IPTG to a final concentration of 1 mM and an additional shaking for 6 h at 37°C. The induced bacteria were pelleted after centrifugation of the 96 deep well block at 2.000 g, resuspended in 100 μl lysis buffer (8 M urea, 50 mM NaH_2_PO_4_, 300 mM NaCl, 10 mM imidazole, pH 8.0), and subsequently cleared by utilizing a Millipore 96-well filtration (clearing) plate (Millipore, Germany) according to the manufacturers' instructions. The cleared bacterial lysates were analyzed by SDS/PAGE followed by Western blotting using the monoclonal mouse anti RGS-His antibody (Qiagen, Germany) for the presence of recombinant, His-tagged proteins.

For fast small scale recombinant protein purification, the bacterial pools that have been detected positive for the presence of His-tagged VZV-proteins were induced in individual 5 ml batch cultures for 6 h at 37°C. Protein purification under denaturating conditions was performed using Ni-NTA spin columns (Qiagen, Germany) according to the manufacturers' instructions. Protein purification was verified by SDS/PAGE following Coomassie staining. Purified His-tagged proteins were stored at -20°C until further usage.

### Serum profiling experiments in microarray format

For serum profiling experiments, the small scale purified, His-tagged VZV proteins were spotted on microarrays. Microarrays (*recom*Dot, Mikrogen, Germany) were processed according to the manufacturer's instructions. Briefly, purified recombinant proteins and VZV lysate (Virion/Serion, Germany) as a control were spotted on nitrocellulose microarrays with a capacity for 34 spots (*recom*Dot system, Mikrogen, Germany). All antigens were spotted in duplicate. Arrays were incubated with 2 ml diluted serum (1:40 in *recom*Dot buffer, Mikrogen, Germany). For detection of specifically bound antibodies anti-human IgG- and anti-human IgM-peroxidase conjugates (Seramun, Germany) were used. Visualization of immune complexes was done with tetramethylbenzidine (TMB) colorization substrate (see Additional file 1, I). After scanning and digitalization, the quantification of specific signals was done with the *recom*Dot Scan software (*recom*Dot system, Mikrogen, Germany). In order to identify putative serological VZV candidate antigens, the microarrays were probed with different clinically and serologically defined patient sera. Clinically defined serum samples were derived from patients suffering from acute varicella or zoster. Serologically defined serum samples (e.g. VZV-IgG negative samples) were pre-analyzed for the presence of VZV-IgG by the commercially available Enzygnost wcELISA (Dade Behring, Germany). All clinically defined serum samples were additionally chracterized for their VZV-IgG and VZV-IgM titers by Enzygnost wcELISA (Dade Behring, Germany).

### Cloning, recombinant expression and purification of selected VZV proteins

Cloning and expression of the selected VZV proteins (ORFs 1, 4, 14, 49 and 68) was performed as described previously [32,33]. The recombinant antigens were expressed in *E. coli *as full-length proteins, with exception of the glycoproteins, which were cloned without transmembrane domains. The expressed proteins were purified to high purity by standard chromatographic methods as described before [12].

### Generation and validation of a recomLine VZV assay

Individual dilutions of the purified recombinant antigens were applied directly onto nitrocellulose membranes in different lines. The appropriate line conditions for all recombinant antigens were determined empirically with standard serum samples. Membranes were blocked with 1% skim milk solution in phosphate-buffered saline, air dried, and cut into individual test strips. Strips were stored at 4°C. Processing of nitrocellulose test strips was performed following the instruction manual for recomLine EBV (Mikrogen, Germany) using the reagents supplied in the kit. Briefly, serum samples were applied at 1:100 dilutions and incubated together with the nitrocellulose test strips for 1 h at room temperature. Following three washing steps of 5 min each, a second incubation of 45 min with peroxidase-labelled secondary antibody (anti human IgG or IgM) was performed. Strips were stained for about 8 min using tetramethylbenzidine after three additional washing steps of 5 min each. Controls were used as described previously [32,33]. The scanner OpticPro S28 (Plustek, Korea) and recomScan software (Mikrogen, Germany) were used according to the manufacture's instructions. The test interpretation may also be easily done manually by direct comparison with the cut-off band provided on the strip.

## Competing interests

MGVP, TJ, AL, JH, HN, GJ and AB have non-financial competing interests.

KIP, CN and MS have received salaries from Mikrogen GmBH.

## Authors' contributions

MGVP carried out the recombinatorial cloning, sequence analysis, expression and purification of proteins in small format, ELISAs assays, data analysis and wrote the manuscript. KIP carried out large scale protein purification, microarray screen, line development and data analysis, participated in the coordination of the study and help to draft the manuscript. TJ, CN and MS carried out large scale protein purification, microarray screen, line development and validation of VZV antigens in Line format. AL, JH, HN and GJ participated in the design of the study. AB conceived the study, and participated in its design and coordination and wrote the manuscript. All authors read and approved the final manuscript.

## Supplementary Material

Additional file 1 **Systematic pipeline for the identification of novel serological markers of VZV infection**. A) Nested PCR for the amplification of VZV ORFs with attB sites, B) BP reaction into pDONR207, C) Characterization of resulting pENTR207 vectors by *BanII *restriction analysis and sequencing, D) LR reaction to insert the customized bacterial expression vector pETG-A-His-N- [rfB], E) Characterization of resulting pETG-A-His-N-VZV-ORF vectors by *Hind*III and *Xba*I restriction analysis and sequencing, F) Transfection of expression vectors into *E. coli *Rosetta (DE3) and induction of protein expression with IPTG, G) Analysis of protein expression by Western blotting using an anti-RGS-His antibody, H) Analysis of purified proteins by SDS-PAGE and Coomassie staining, I) Screening for serologically reactive antigens in microarray format, J) Rearrangement of screened marker antigens in Line format. Click here for file

Additional file 2 **Validation of the VZV RecomLine with clinically defined serum samples**. This table depicts all serological parameters of the clinically defined patient sera.VZV-IgG and IgM ELISA titres were assayed by wcELISA (Dade Behring, Enzygnost, Germany). Qualitative RecomLine VZV IgG and IgM reactivities towards individual antigens are depicted as "1" (reactive) and "0" (non reactive). Click here for file

Additional file 3 **Analysis of possible crossreactivities of the RecomLine VZV recombinant antigens with serum samples of defined antibody status to HSV**. This table depicts the cross-reactivity of all serologically defined VZV-IgG negative patient samples according to their HSV (IgG) status. No cross-reactivity in the in the RecomLine VZV IgM assay can be observed. The recomLine VZV IgG assay exhibits some reactivities against ORFs 4, 14, 49 but not against ORF68. No correlation of cross-reactivities and HSV-1 status can be observed. Click here for file
