# Supplementary material for: A systematic approach for the identification of novel, serologically reactive recombinant Varicella-Zoster Virus (VZV) antigens
Source: Virol J. 2010 Jul 20;7:165. doi: 10.1186/1743-422X-7-165 (PMC2915977; doi:10.1186/1743-422X-7-165)
Supplement: Additional file 1 — Systematic pipeline for the identification of novel serological markers of VZV infection. A) Nested PCR for the amplification of VZV ORFs with attB sites, B) BP reaction into pDONR207, C) Characterization of resulting pENTR207 vectors by BanII restriction analysis and sequencing, D) LR reaction to insert the customized bacterial expression vector pETG-A-His-N- [rfB], E) Characterization of resulting pETG-A-His-N-VZV-ORF vectors by HindIII and XbaI restriction analysis and sequencing, F) Transfection of expression vectors into E. coli Rosetta (DE3) and induction of protein expression with IPTG, G) Analysis of protein expression by Western blotting using an anti-RGS-His antibody, H) Analysis of purified proteins by SDS-PAGE and Coomassie staining, I) Screening for serologically reactive antigens in microarray format, J) Rearrangement of screened marker antigens in Line format. [file 1743-422X-7-165-S1.PDF]

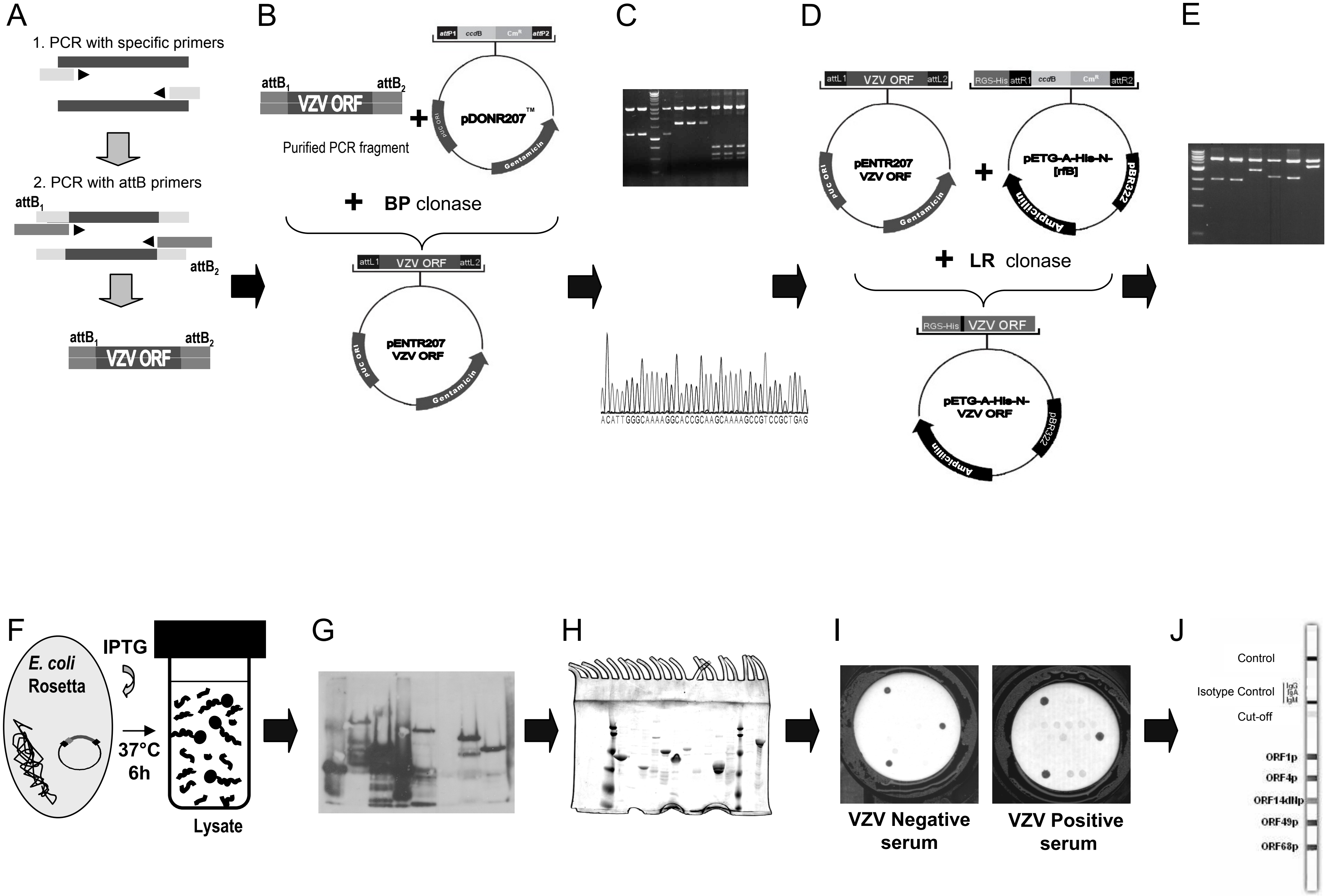

Supplementary Figure 1

Supplementary Figure 1. Systematic pipeline for the identification of novel serological markers of VZV infection. A) Nested PCR for the amplification of VZV ORFs with attB sites, B) BP reaction into pDONR207, C) Characterization of resulting pENTR207 vectors by *BanII* restriction analysis and sequencing, D) LR reaction to insert the VZV ORF into the customized bacterial expression vector pETG-A-His-N-[rfB], E) Characterization of resulting pETG-A-His-N-VZV-ORF vectors by *Hind*III and *Xba*I restriction analysis and sequencing, F) Transfection of expression vectors into *E. coli* Rosetta (DE3) and induction of protein expression with IPTG, G) Analysis of protein expression by Western blotting using an anti-RGS-His antibody, H) Analysis of purified proteins by SDS-PAGE and Coomassie staining, I) Screening for serologically reactive antigens in microarray format, J) Rearrangement of screened marker antigens in Line format.
