# Supplementary material for: A systematic approach for the identification of novel, serologically reactive recombinant Varicella-Zoster Virus (VZV) antigens
Source: Virol J. 2010 Jul 20;7:165. doi: 10.1186/1743-422X-7-165 (PMC2915977; doi:10.1186/1743-422X-7-165)
Supplement: Additional file 2 — Validation of the VZV RecomLine with clinically defined serum samples. This table depicts all serological parameters of the clinically defined patient sera.VZV-IgG and IgM ELISA titres were assayed by wcELISA (Dade Behring, Enzygnost, Germany). Qualitative RecomLine VZV IgG and IgM reactivities towards individual antigens are depicted as "1" (reactive) and "0" (non reactive). [file 1743-422X-7-165-S2.PDF]

Supplementary Table 1. Validation of the VZV RecomLine with clinically defined serum samples

Sample Code	Diagnosis	wcELISA		N° Tests	RecomLine IgM (ORFs) reactivities					N° Tests	RecomLine IgG (ORFs) reactivities				
		IgM	IgG (IU/l)		1	4	14	49	68		1	4	14	49	68
CP1	CP	1:160	27	1	0	0	1	0	1	1	0	1	1	1	1
CP2	CP	>1:160	25	1	0	1	1	1	1	1	0	0	1	1	1
CP3	CP	>1:160	22	1	0	1	1	1	1	1	0	1	1	1	1
CP4	CP	1:40	2,2	1	0	1	1	0	1	1	0	1	1	1	1
CP5	CP	1:160	26,0	1	0	0	0	0	1	1	0	0	1	1	1
**Total N° of samples**				**5**	**0**	**3**	**4**	**2**	**5**	**5**	**0**	**3**	**5**	**5**	**5**
**%**				**100,00**	**0,00**	**60,00**	**80,00**	**40,00**	**100,00**	**100,00**	**0,00**	**60,00**	**100,00**	**100,00**	**100,00**
HZ1	HZ	1:40	23	1	0	0	0	0	1	1	0	0	1	1	1
HZ2	HZ	1:40	23	1	0	0	0	0	1	1	0	0	1	1	1
HZ3	HZ	1:160	21	1	0	0	0	0	1	1	0	1	0	1	1
HZ4	HZ	1:160	21	1	0	0	0	0	1	1	0	1	1	1	1
HZ5	HZ	1:160	21	1	0	0	0	0	1	1	0	1	0	1	1
HZ6	HZ	1:40	16	1	0	0	0	0	1	1	0	1	1	1	1
HZ7	HZ	Negative	11	1	0	0	0	0	0	1	0	0	0	0	1
HZ8	HZ	Negative	19	1	0	0	0	0	1	1	0	0	0	0	1
HZ9	HZ	1:40	29	1	0	0	0	0	1	1	0	0	1	1	1
HZ10	HZ	1:40	16	1	0	0	0	0	1	1	0	1	1	1	1
HZ11	HZ	Negative	Positive*	1	0	0	0	0	0	1	1	1	1	1	1
HZ12	HZ	Negative	Positive*	1	0	0	0	0	0	1	0	1	0	1	1
HZ13	HZ	Negative	Positive*	1	0	0	0	0	0	1	1	0	0	0	1
HZ14	HZ	Negative	Positive*	1	0	0	0	0	0	1	1		1	0	0
HZ15	HZ	Negative	Positive*	1	0	0	0	0	0	1	1	1	1	1	1
HZ16	HZ	Positive*	Positive*	1	0	0	0	0	1	1	1	0	1	1	1
HZ17	HZ	Negative	Positive*	1	0	0	0	0	0	1	1	0	0	1	1
HZ18	HZ	Negative	Positive*	1	0	0	0	0	1	1	0	0	1	1	1
**Total N° of samples**				**18**	**0**	**0**	**0**	**0**	**11**	**18**	**6**	**8**	**11**	**14**	**17**
**%**				**100**	**0,00**	**0,00**	**0,00**	**0,00**	**61,11**	**100,00**	**33,33**	**44,44**	**61,11**	**77,78**	**94,44**

* Titres not available
CP= chickenpox
HZ= Herpes-Zoster

Supplementary Table 1. This table depicts all serological parameters of the clinically defined patient sera. VZV-IgG and IgM ELISA titres were assayed by wcELISA (Dade Behring, Enzygnost, Germany). Qualitative RecomLine VZV IgG and IgM reactivities towards individual antigens are depicted as "1" (reactive) and "0" (non reactive).
