# Supplementary material for: A systematic approach for the identification of novel, serologically reactive recombinant Varicella-Zoster Virus (VZV) antigens
Source: Virol J. 2010 Jul 20;7:165. doi: 10.1186/1743-422X-7-165 (PMC2915977; doi:10.1186/1743-422X-7-165)
Supplement: Additional file 3 — Analysis of possible crossreactivities of the RecomLine VZV recombinant antigens with serum samples of defined antibody status to HSV. This table depicts the cross-reactivity of all serologically defined VZV-IgG negative patient samples according to their HSV (IgG) status. No cross-reactivity in the in the RecomLine VZV IgM assay can be observed. The recomLine VZV IgG assay exhibits some reactivities against ORFs 4, 14, 49 but not against ORF68. No correlation of cross-reactivities and HSV-1 status can be observed. [file 1743-422X-7-165-S3.PDF]

Supplementary Table 2. Analysis of possible cross-reactivities of the RecomLine VZV recombinant antigens with serum samples of defined antibody status to HSV

Sample Code	HSV-Status*	VZV-Status**	N° Tests	RecomLine IgM (ORFs) reactivities				N° Tests	RecomLine IgG (ORFs) reactivities				
				4	14	49	68		1	4	14	49	68
HN1	Negative	Negative	1	0	0	0	0	1	0	0	0	0	0
HN2	Negative	Negative	1	0	0	0	0	1	0	0	1	1	0
HN3	Negative	Negative	1	0	0	0	0	1	0	0	0	0	0
HN4	Negative	Negative	1	0	0	0	0	1	0	1	0	1	0
HN5	Negative	Negative	1	0	0	0	0	1	0	0	0	0	0
HN6	Negative	Negative	1	0	0	0	0	1	0	0	0	0	0
HN7	Negative	Negative	1	0	0	0	0	1	0	0	1	1	0
HN8	Negative	Negative	1	0	0	0	0	1	0	0	0	0	0
HN9	Negative	Negative	1	0	0	0	0	1	0	0	0	0	0
**Total N° of samples**			**9**	**0**	**0**	**0**	**0**	**9**	**0**	**1**	**2**	**3**	**0**
**%**			**100,00**	**0,00**	**0,00**	**0,00**	**0,00**	**100,00**	**0,00**	**11,11**	**22,22**	**33,33**	**0,00**
HP1	Positive	Negative	1	0	0	0	0	1	0	0	0	0	0
HP2	Positive	Negative	1	0	0	0	0	1	0	0	0	0	0
HP3	Positive	Negative	1	0	0	0	0	1	0	0	0	0	0
HP4	Positive	Negative	1	0	0	0	0	1	0	0	0	0	0
HP5	Positive	Negative	1	0	0	0	0	1	0	0	0	0	0
HP6	Positive	Negative	1	0	0	0	0	1	0	0	0	0	0
HP7	Positive	Negative	1	0	0	0	0	1	0	0	0	0	0
HP8	Positive	Negative	1	0	0	0	0	1	0	0	0	0	0
HP9	Positive	Negative	1	0	0	0	0	1	0	0	0	0	0
HP10	Positive	Negative	1	0	0	0	0	1	0	0	0	0	0
HP11	Positive	Negative	1	0	0	0	0	1	0	0	0	0	0
HP12	Positive	Negative	1	0	0	0	0	1	0	0	0	0	0
HP13	Positive	Negative	1	0	0	0	0	1	0	0	0	0	0
HP14	Positive	Negative	1	0	0	0	0	1	0	0	0	1	0
HP15	Positive	Negative	1	0	0	0	0	1	0	0	1	0	0
**Total N° of samples**			**15**	**0**	**0**	**0**	**0**	**15**	**0**	**0**	**1**	**1**	**0**
**%**			**100,00**	**0,00**	**0,00**	**0,00**	**0,00**	**100,00**	**0,00**	**0,00**	**6,67**	**6,67**	**0,00**

*= Serological status according to HSV ELISA (Dade Behring, Enzygnost, Germany)
**= Serological status according to wcVZV ELISA (Dade Behring, Enzygnost, Germany)
N°= number

Supplementary Table 2. This table depicts the cross-reactivity of all serologically defined VZV-IgG negative patient samples according to their HSV (IgG) status. No cross-reactivity in the Recomline VZV IgM assay can be observed . The recomLine VZV IgG assay exhibits some reactivities against ORFs 4, 14 and 49 but not against ORF68. No correlation of cross-reactivities and HSV-IgG status can be observed.
